# Relationship Between Image Quality and Bias in 3D Echocardiographic Measures: Data From the SABRE (Southall and Brent Revisited) Study

**DOI:** 10.1161/JAHA.120.019183

**Published:** 2022-04-27

**Authors:** Lamia Al Saikhan, Chloe Park, Therese Tillin, Guy Lloyd, Jamil Mayet, Nish Chaturvedi, Alun D. Hughes

**Affiliations:** ^1^ Department of Cardiac Technology College of Applied Medial Sciences Imam Abdulrahman Bin Faisal University Dammam Kingdom of Saudi Arabia; ^2^ MRC Unit for Lifelong Health and Ageing Department of Population Science & Experimental Medicine UCL Institute of Cardiovascular Science University College London London United Kingdom; ^3^ Department of Cardiovascular Imaging Barts Heart Centre Barts Health NHS Trust London United Kingdom; ^4^ NIHR Imperial Biomedical Research Centre Imperial College London and Imperial College Healthcare NHS Trust Hammersmith Hospital London United Kingdom

**Keywords:** 3D echocardiography, image quality, left ventricle, myocardium, speckle‐tracking, Echocardiography

## Abstract

**Background:**

Image‐quality (IQ) compromises left ventricle assessment by 3‐dimensional echocardiography (3DE). Sicker/frailer patients often have suboptimal IQ, and therefore observed associations may be biased by IQ. We investigated its effect in an observational study of older people and when IQ was modified experimentally in healthy volunteers.

**Methods and Results:**

3DE feasibility by IQ was assessed in 1294 individuals who attended the second wave of the Southall and Brent Revisited study and was compared with 2‐dimensional (2D)‐echocardiography feasibility in 147 individuals. Upon successful analysis, means of ejection fraction (3D‐EF) and global longitudinal strain (3D‐GLS) (plus 2D‐EF) were compared in individuals with poor versus good IQ. In 2 studies of healthy participants, 3DE‐IQ was impaired by (1) intentionally poor echocardiographic technique, and (2) use of a sheet of ultrasound‐attenuating material (neoprene rubber; 2–4 mm). The feasibility was 41% (529/1294) for 3DE versus 61% (89/147) for 2D‐EF, *P*<0.0001. Among acceptable images (n=529), good IQ by the 2015 American Society of Echocardiography/European Association of Cardiovascular Imaging criteria was 33.6% (178/529) and 71.3% (377/529) for 3D‐EF and 3D‐GLS, respectively. Individuals with poor IQ had lower 3D‐EF and 3D‐GLS (absolute) than those with good IQ (3D‐EF: 52.8±6.0% versus 55.7±5.7%, Mean‐Δ −2.9 [−3.9, 1.8]; 3D‐GLS: 18.6±3.2% versus 19.2±2.9%, Mean‐Δ −0.6 [−1.1, 0.0]). In 2 experimental models of poor IQ (n=36 for both), mean differences were (−2.6 to −3.2) for 3D‐EF and (−1.2 to −2.0) for 3D‐GLS. Similar findings were found for other 3DE left ventricle volumes and strain parameters.

**Conclusions:**

3DE parameters have low feasibility and values are systematically lower in individuals with poor IQ. Although 3D‐EF and 3D‐GLS have potential advantages over conventional echocardiography, further technical improvements are required to improve the utility of 3DE in clinical practice.

**Nonstandard Abbreviations and Acronyms**
3DE: 3D echocardiography
GLS: global longitudinal strain
IQ: image quality
STE: speckle‐tracking echocardiography

**Clinical Perspective**

**What Is New?**

- Three‐dimensional echocardiographic analysis of left ventricle including parameters such as ejection fraction and global longitudinal strain have low feasibility, and when feasible, values of ejection fraction and deformations are systematically lower in individuals with poorer image quality.

**What Are the Clinical Implications?**

- Although ejection fraction and global longitudinal strain by means of transthoracic 3D echocardiography have potential advantages over 2D echocardiography, further technical improvements may be required to improve the utility of 3D echocardiography in clinical practice.

Accurate assessment of left ventricular (LV) function by echocardiography is important for the determination of prognosis and therapeutic strategies.^1^ Recently, 3‐dimensional echocardiography (3DE) and speckle‐tracking echocardiography (STE) have emerged as a promising tools to quantify myocardial performance.^2^ To date most STE studies have used 2‐dimensional STE (2D‐STE),^3^, ^4^ but 3‐dimensional STE (3D‐STE) may overcome some of the limitations of 2D‐STE, such as “out of plane” motion, and variability due to nonsimultaneous acquisitions^2^; however, the comparatively low spatial and temporal resolution of 3D‐STE is a concern.^5^

Image quality (IQ) is expected to influence 3DE and STE‐derived indices,^2^, ^6^, ^7^ but quantitative evidence on the extent to which IQ influences measures of myocardial mechanics is limited. This is important because sicker/frailer patients often have suboptimal echocardiographic IQ and therefore observed associations may be biased by IQ. Previous studies have either measured associations between 3D‐STE LV deformation indices and IQ after excluding unhealthy individuals^7^ or evaluated the impact of IQ by excluding individuals with suboptimal images,^6^, ^8^ but both approaches will introduce selection bias.

We therefore aimed to measure associations between 3DE‐derived LV myocardial indices and IQ controlled for potential confounders in a large sample of community‐dwelling individuals (the SABRE [Southall and Brent Revisited] study)^9^ and compared estimates of bias with experimental studies that intentionally impaired IQ.

## METHODS

### Study Populations

#### Observational Study

In the SABRE study 1438 participants underwent comprehensive examinations including transthoracic echocardiography, anthropometry, ECG, and blood pressure. In brief, SABRE is a UK triethnic population‐based longitudinal cohort (age at second wave of follow‐up: 69.6±6.2 years).^9^, ^10^ The study was approved by St Mary’s Hospital Local Research Ethics Committee (07/H0712/109), and written informed consent was obtained.

#### Experimental Studies

Young healthy volunteers with excellent echocardiographic windows were recruited. Height, weight, and sitting resting blood pressure were measured. IQ was impaired using 2 approaches: intentionally poor image acquisition technique and impairing ultrasound propagation using an attenuating material, analogous to an unfavorable body habitus (neoprene study). These protocols were approved by University College London Local Research Ethics Committee and written informed consent was obtained. Further details regarding SABRE can be found at https://mrc.ukri.org/research/facilities‐and‐resources‐for‐researchers/cohort‐directory/southall‐and‐brent‐revisited‐sabre/. Because of the sensitive nature of the data collected for this study, requests to access the data set from qualified researchers trained in human subject confidentiality protocols may be sent to the MRC Unit for Lifelong Health and Ageing at University College London (sabre@ucl.ac.uk).

### Imaging

Imaging in SABRE was performed by 2 experienced cardiac sonographers in accordance with American Society of Echocardiography (ASE) guidelines,^11^ using a Phillips iE33 ultrasound machine equipped with a S5‐1 phased‐array ultrasound transducer and a matrix array (X3‐1) transducer. The SABRE echocardiography imaging protocol, including feasibility of conventional echocardiography, has been described previously, but Table S1 shows those results relevant to this study.^12^ Briefly, 3DE full‐volume LV data sets of 4 subvolumes acquired over 4 cardiac cycles during held respiration and in a wide‐angled mode (93°×80°) were obtained from the apical window. Depth, sector width, and gain settings were adjusted appropriately.^11^

To assess the feasibility of 3DE based on IQ, the following were excluded from the denominator: participants who attended the clinic before the availability of 3D probe (n=37), in atrial fibrillation (n=25) or with inadequate ECG signal (n=3), operator deviations from the protocol or other technical nonimaging reasons (eg, frame rate set too low, images missing; n=79) leaving a total denominator N=1294 (Figure).

Imaging for the experimental studies was performed by a single sonographer using a Philips EPIQ‐7 ultrasound machine equipped with Xmatrix‐array transducer (X5‐1). Participants were scanned using a standard protocol.^11^, ^13^, ^14^ Harmonic imaging and multiple‐beat 3DE mode were used; 4 wedge‐shaped subvolumes were acquired over 4 cardiac cycles during a single breath‐hold. Care was taken to include the entire LV cavity within the pyramidal sector volume.

In the “poor technique” study, 2 gated wide‐angled 3DE full‐volume data sets were obtained per participant from the apical window. The first acquisition was performed according to European Association of Echocardiography/ASE guidelines.^11^ Machine settings were adjusted to optimize the IQ ensuring clear visualization of LV endocardial borders and avoiding echo dropout. A good 3DE image was defined as clear visualization of the endocardium in all 16 segments in both end‐diastolic and end‐systolic frames. The second acquisition was captured after intentionally impairing the IQ with suboptimal echo technique. This was achieved by supine scanning and omission of gel to create an air‐tissue interface initiating multiple reflections and acoustic shadowing artifacts. A suboptimal 3DE image was defined as the presence of at least 1 of the following (Figure S1A): (1) poor visualization of the endocardium throughout the cardiac cycle in up to 7 segments, (2) the presence of echo dropout, and (3) shadow artifacts. The acquisition protocol was repeated on the same day to assess the test‐retest reproducibility.

In the neoprene study, the quality of the 3DE images was impaired in a graded and controlled manner by placing a sheet of ultrasound‐attenuating material, neoprene, of 3 different thicknesses (2, 3, and 4 mm) to mimic mildly, moderately, and severely impaired IQ, respectively (Figure S1B) between the skin and the transducer with ultrasound gel on both sides. Neoprene was chosen as many of its acoustic properties are similar to soft biological tissues, it is durable, and it has a comparatively high attenuation coefficient.^15^ Four gated 3DE full‐volume data sets were acquired per participant. All acquisitions were free of stitching artifacts with good quality ECG signals. The best frame rate was established for each individual under optimal conditions and was maintained constant throughout the study with a minimum acceptable acquisition rate of 18 frames per second (Hz).^5^

### Image Analysis

All conventional echocardiographic analyses in SABRE study were performed on the ultrasound machine during the clinic visit using Philips QLAB software 7.0, averaging 3 measurements.^12^ LV dimensions and wall thickness from 2D‐guided M‐mode were measured from the parasternal long‐axis view from which LV mass was calculated, following the ASE recommendations.^16^ LV volumes from conventional 2D‐echocardiography were calculated by the Teichholz formula using the linear dimensions from which LV ejection fraction (EF) was derived to maintain the compatibility with previous sweeps and permit comparisons with other cohort studies.^16^ Tissue Doppler analysis of lateral and septal mitral annulus motion and mitral inflow analysis by PW Doppler were performed for LV diastolic function assessment.^17^

LV 3D images were analyzed using 4D LV‐Analysis software (TomTec Imaging Systems GmbH) by a single experienced reader and manual adjustments of the endocardial border were minimized (as described in Data S1). The 4D LV‐Analysis calculates imaging rates as frames per cardiac cycle rather than per second; therefore, using a constant acquisition rate (Hz) may result in differing rates per cycle due to variations in heart rate.

There is no uniform standard for grading LV 3D images. In SABRE, IQ was routinely assessed as:

1. Good (score‐1)=clear visualization of endocardium in all 16 segments.
2. Fair (score‐2)=unclear visualization of endocardium in ≤2 segments or presence of minor artifacts, for example, apical noise.
3. Adequate (score‐3)=unclear visualization of endocardium in ≤6 segments.
4. Poor (score‐4)=unclear visualization of endocardium in >6 segments, but reliable tracking throughout the cardiac cycle using the adjacent segments as a reference.
5. Unacceptable IQ was defined as presence of major stitching artifacts preventing reliable tracking of the endocardium, unacceptable visualization of the LV endocardial boundaries, or ≥4 segments being outside the image sector.^16^

The SABRE IQ score was modified slightly when grading LV apical 2D images for 2D‐EF to only 12 segments in total instead of 16 segments (ie, 6 segments per each apical view).

To allow comparison with other image‐scoring schemes in the literature and to examine the sensitivity to the SABRE quality grading system employed, 2 other grading systems were used.

The first was according to the 2015 ASE/European Association of Cardiovascular Imaging (EACVI) guidelines for chamber quantification.^16^, ^18^ For LV 2D‐ and 3D‐EF (full volume method), “poor” IQ was defined as ≥2 contiguous segments with inadequate endocardial delineation and for 3D global longitudinal strain (3D‐GLS, STE method), “poor” IQ was defined as >2 segments with inadequate endocardial delineation in any LV apical view.

The second image scoring system (poor IQ segments score)^19^ used 4 categories based on number of poor segments: none, 1 segment, 2 segments, and ≥3‐segments (contiguous for 2D‐ and 3D‐EF and in any apical view for 3D‐GLS). Feasibility of 3D‐EF was compared with LV EF by 2D echocardiography using the biplane method of disks (modified Simpson’s rule) in 147 participants from the SABRE cohort. Both grading systems (ie, the 2015 ASE/EACVI guidelines‐based IQ score and the poor IQ segments score) were used when the quality of LV apical 2D images was assessed to obtain 2D‐EF measurements from apical 4‐ and 2‐chamber views.

Primary indices for 3DE were 3D‐EF and 3D‐GLS as these are commonly used in clinical practice. All 3DE LV deformation indices (strains and rotations) were presented as absolute values to facilitate interpretation. Additional 3DE LV myocardial indices were (1) volumes (end‐diastolic, end‐systolic, and stroke volumes); (2) LV rotational indices (basal and apical rotations, twist, and torsion); and (3) LV global circumferential strain and peak averaged segmental strains (longitudinal, circumferential, radial, and principal tangential strains [a fuller description of segmental myocardial deformation incorporating both longitudinal and circumferential strain]). Peak averaged segmental strain measures were calculated as the average of the individual 16‐segment values. Global strain measures were computed based on the entire contour length of longitudes (ie, averaged over the myocardium). Reproducibility of LV myocardial indices by means of transthoracic 3DE in SABRE population has been reported previously.^12^

### Statistical Analysis

All analyses were performed using STATA (15.1, StataCorp LLC). Sample data are summarized as mean±SD or counts (percentages) for continuous and categorical variables, respectively.

Differences in continuous variables between 2 groups were assessed using a 2‐sample *t* test (with Welch's correction for unequal variance if necessary), and ANOVA for more than 2 groups, and a χ^2^ test for categorical variables. Nonparametric tests (Wilcoxon or Kruskal‐Wallis) were used if the data did not meet the assumptions of normality or homogeneity of variance for parametric tests. Estimated population means and dispersion of LV myocardial indices by IQ scores are presented as mean±SD (or median [interquartile range]) and mean differences (95% CI).

Multiple linear regression was performed to quantify associations between IQ scores or frames/cycle and LV myocardial indices after adjustment for confounders selected a priori: age, sex, ethnicity, height, weight, heart rate, and history of percutaneous coronary intervention and/or coronary artery bypass graft and/or chronic obstructive pulmonary disease. Regression model diagnostics were performed ensuring all assumptions of multiple linear regression were satisfied. To permit comparison of the magnitude of adjusted bias from the observational study with the experimentally induced bias, data were normalized to the overall mean of indices (% absolute standardized bias=regression coefficient/overall mean). We also assessed whether abnormal 3D‐EF, (ie, <50%), modified associations between IQ scores and LV myocardial indices (ie, creating worse bias than normal 3D‐EF).

For the experimental studies, systematic differences in LV myocardial indices due to IQ were assessed using mixed linear models with participant ID as a random effect and quality and scan replicate number as fixed effects. Data were normalized to the mean of good quality images to permit comparison of magnitude of bias across indices (% absolute standardized bias). In the poor technique study, test‐retest/scan‐rescan reliability was summarized using an intraclass correlation coefficient (ICC) estimated using mixed linear models and categorized as follows: ICC<0.4=poor, 0.4≥ICC<0.75=fair to good, and ICC ≥0.75=excellent.^20^ Test‐retest reproducibility was also assessed using Bland‐Altman plots and summarized as mean differences (limits of agreement). Rereading the same (good quality) scans was also performed blinded to the original measurements after 2 to 3 months interval. A 2‐tailed *P* value of <0.05 was considered statistically significant.

For the comparison of feasibility of EF by 3DE and 2D echocardiography, a sample size calculation was performed to determine the number of participants with 2D‐EF analysis needed to detect a difference of 14% with 90% power with a 2‐sided alpha of 0.05; this was 147.

For the experimental studies, the sample size was chosen to ensure a lower limit of the 1‐sided CI of the ICC ≤0.15. This also enabled detection of a bias ≥1 SD (α=0.05) with 96% power.

## RESULTS

### Study Population

Characteristics of the participants in the observational (SABRE) and experimental studies are shown in Tables 1 and 2, respectively.

**Table 1:** Characteristics of SABRE Participants in Whom 3DE was Feasible (n=529)

Age, y	69.1±6.1
Male sex, n (%)	405 (77)
Ethnicity, European/South Asian/African Caribbean (%)	52/28/20
Systolic blood pressure, mm Hg	140.2±17.9
Diastolic blood pressure, mm Hg	76.5±9.6
Heart rate, bpm	67.2±11.4
Body mass index, kg/m^2^	26.1±3.5
Waist:hip ratio	0.96±0.07
Hypertension, n (%)	301 (56.9)
Diabetes, n (%)	118 (22.3)
Prior coronary heart disease, n (%)	89 (16.8)
Smoking status, % never/ex/current	54.1/38.1/7.8
3DE‐derived left ventricular myocardial indices	
EF, %	53.8±6.0
EF <50%, n (%)	90 (17%)
End‐diastolic volume, mL/m^2^	57.3±13.3
End‐systolic volume, mL/m^2^	26.7±8.4
Stroke volume, mL	56.5±14.1
Global CS, %	−25.6±4.1
Global LS, %	−19.0±3.0
Peak averaged CS, %	−25.8±4.1
Peak averaged LS, %	−18.3±2.9
Peak averaged principal tangential strain, %	−31.1±4.1
Peak averaged radial strain, %	37.0±5.2
Peak basal rotation, °	−5.4±3.3
Peak apical rotation, °	8.3±4.4
Peak twist, °	13.4±6.9
Peak torsion, °/cm	1.7±0.9

Data are mean± SD or n (%). 3DE indicates 3‐dimensional echocardiography; CS, circumferential strain; LS, longitudinal strain; and SABRE, Southall and Brent Revisited study.

**Table 2:** Characteristics of Participants in the Experimental Studies

	Poor technique (n=18)	Neoprene (n=18)
Age, y	28±6	31±6
Male sex, n (%)	10 (55.5%)	15 (83.3%)
Systolic blood pressure, mm Hg	118.2±8.6	123.2±9.2
Diastolic blood pressure, mm Hg	73.5±7.3	77.3±9.5
Heart rate, bpm	72±13.8	69.1±14.2
Height, cm	169.9±9.4	172.2±8.7
Weight, kg	70.9±16.1	73.0±8.1

Data are mean±SD or n (%).

### Feasibility and Quality of 3DE in SABRE

From a total sample size of 1438, 144 participants were excluded for various nonimaging reasons and there were 529 participants in whom 3DE was successful (Figure). The feasibility of 3DE based on IQ (ie, excluding nonimaging reasons) was 41% (529/1294), whereas the feasibility of 2D‐EF analysis was 61% (89/147), *P*<0.0001 for comparison. In those individuals (n=529), the prevalence of good IQ defined using the 2015 ASE/EACVI criteria was 33.6% (178 out of 529) for 3D‐EF and 71.3% (377 out of 529) for 3D‐GLS (Tables 3 and 4). The other more graded scoring methods gave broadly similar results (Tables 3 and 4). By contrast, the prevalence of good IQ defined using the 2015 ASE/EACVI criteria was 69.7% (62 out of 89) for 2D‐EF being higher than 3D‐EF (Table 5). The other more graded scoring methods gave broadly similar results for 2D‐EF (Table 5).

**Table 3:** Comparison 3D‐EF by Different Image‐Quality Scores in the SABRE Study (n=529)

2015 ASE/EACVI guidelines‐based image‐quality score			
	Good	Poor	*P* value
n (%)	178 (33.6)	351 (66.4)	
Mean±SD, %	55.7±5.7	52.8±6.0	<0.0001
Mean Δ (95% CI), %	Reference	−2.9 (−3.9 to −1.8)	

Poor image‐quality segments score					
	None‐segment	1 segment	2 segments	≥3 segments	*P* value
n (%)	63 (11.9)	115 (21.7)	219 (41.4)	132 (25.0)	
Mean±SD, %	56.4±4.8	55.2±6.0	52.8±6.2	52.8±5.6	<0.0001
Mean Δ (95% CI), %	Reference	−1.2 (−3.0 to 0.6)	−3.6 (−5.3 to −2.0)	−3.5 (−5.3 to −1.8)	

SABRE image‐quality score					
	Good	Fair	Adequate	Poor	*P* value
n (%)	19 (3.6)	235 (44.4)	239 (45.2)	36 (6.8)	
Mean±SD, %	56.5±4.8	55.2±5.7	52.3±6.1	52.6±5.3	<0.0001
Mean Δ (95% CI), %	Reference	−1.2 (−4.0 to 1.5)	−4.2 (−7.0 to −1.5)	−3.9 (−7.2 to −0.7)	

The 2015 ASE/EACVI guidelines‐based image‐quality score was defined poor for 2‐dimensional‐ and 3D‐EF when ≥2 contiguous segments with inadequate endocardial delineation. 3D‐EF indicates 3‐dimensional ejection fraction; ASE/EACVI, American Society of Echocardiography/European Association of Cardiovascular Imaging; and SABRE, Southall and Brent Revisited study.

**Table 4:** Comparison 3D‐GLS by Different Image‐Quality Scores in the SABRE Study (n=529)

2015 ASE/EACVI guidelines‐based image‐quality score			
	Good	Poor	*P* value
n (%)	377 (71.3)	152 (28.7)	
Mean±SD, %	19.2±2.9	18.6±3.2	0.058
Mean Δ (95% CI), %	Reference	−0.6 (−1.1, 0.0)	

Poor image‐quality segments score					
	None‐segment	1 segment	2 segments	≥3 segments	*P* value
n (%)	63 (11.9)	103 (19.5)	212 (40.1)	151 (28.5)	
Mean±SD,%	19.9±2.7	19.7±2.9	18.7±2.9	18.6±3.2	0.0008
Mean Δ (95% CI), %	Reference	−0.2 (−1.1 to 0.7)	−1.2 (−2.1 to −0.4)	−1.3 (−2.2 to −0.4)	

SABRE image‐quality score					
	Good	Fair	Adequate	Poor	*P* value
n (%)	19 (3.6)	235 (44.4)	239 (45.2)	36 (6.8)	
Mean±SD, %	20.0±2.1	19.4±2.8	18.5±3.2	19.5±3.3	0.004
Mean Δ (95% CI), %	Reference	−0.6 (−2.0 to 0.8)	−1.5 (−2.9 to −0.1)	−0.5 (−2.1 to 1.2)	

The 2015 ASE/EACVI guidelines‐based image‐quality score was defined poor for 3D‐GLS when >2 segments with inadequate endocardial delineation in any left ventricular apical views. 3D‐GLS indicates 3‐dimensional global longitundinal strain; ASE/EACVI, American Society of Echocardiography/European Association of Cardiovascular Imaging; and SABRE, Southall and Brent Revisited study.

**Table 5:** Comparison 2D‐EF by Different Image‐Quality Scores in the SABRE Study (n=89)

2015 ASE/EACVI guidelines‐based image‐quality score			
	Good	Poor	*P* value
n (%)	62 (69.7)	27 (30.3)	
Mean±SD, %	67.1±4.9	61.6±5.0	<0.0001
Mean Δ (95% CI), %	Reference	−5.5 (−7.7, −3.2)	

Poor image‐quality segments score					
	None‐segment	1 segment	2 segments	≥3 segments	*P* value
n (%)	22 (24.7)	40 (44.9)	24 (27.0)	3 (3.4)	
Mean±SD, %	67.8±5.7	66.7±4.4	61.4±5.1	63.5±4.4	0.0001
Mean Δ (95% CI), %	Reference	−1.1 (−3.7 to 1.5)	−6.4 (−9.3 to −3.5)	−4.3 (−10.4 to 1.7)	

Modified SABRE image‐quality score					
	Good	Fair	Adequate	Poor	*P* value
n (%)	22 (24.7)	47 (52.8)	20 (22.5)	0.0 (0.0)	
Mean±SD, %	67.8±5.7	65.8±5.1	62.0±4.6	…	0.0017
Mean Δ (95% CI), %	Reference	−2.0 (−4.6 to 0.67)	−5.8 (−9.0 to −2.6)	…	

The 2015 ASE/EACVI guidelines‐based image‐quality score was defined poor for 2D‐EF and 3‐dimensional‐EF when ≥2 contiguous segments with inadequate endocardial delineation. 2D‐EF indicates 2‐dimensional ejection fraction; ASE/EACVI, American Society of Echocardiography/European Association of Cardiovascular Imaging; and SABRE, Southall and Brent Revisited study.

Participants from whom 3DE LV data could not be acquired or was unacceptable were older, more likely to be South Asian, heavier, and more likely to have hypertension, diabetes, and history of coronary heart disease (Table S2).

### Relationships Between 3D‐EF/3D‐GLS and Image Quality in SABRE

Using the 2015 ASE/EACVI guidelines‐based IQ score, individuals with poor IQ had lower values of 3D‐EF and 3D‐GLS than those with good IQ (3D‐EF: 52.8±6.0% versus 55.7±5.7%; mean differences −2.9 [95% CI, −3.9 to −1.8]; absolute 3D‐GLS: 18.6±3.2% versus 19.2±2.9%; mean differences −0.6 [95% CI, −1.1 to 0.0]; respectively) (Tables 3 and 4). Other IQ scores showed a graded relationship between poorer IQ score and reduced values of 3D‐EF and 3D‐GLS (Tables 3 and 4). The association between poorer IQ, based on all IQ scores, and lower 3D‐EF and 3D‐GLS was preserved even after adjusting for confounders (Table S3). Although the feasibility of 2D‐EF was higher/better than 3D‐EF, individuals with poor IQ, as defined by the 2015 ASE/EACVI guidelines‐based IQ score, also had lower values of 2D‐EF than those with good IQ (2D‐EF: 61.6±5.0% versus 67.1±4.9%; mean differences −5.5 [95% CI, −7.7 to −3.2]; Table 5). Other IQ scores showed a graded relationship between poorer IQ score and reduced values of 2D‐EF.

Similar evidence of graded bias related to IQ was found for 3DE‐derived LV volumes and other LV strain and rotational indices including global circumferential and radial strain and LV twist and torsion, using the poor IQ segments and SABRE IQ scores (Tables S4 and S5). The association between poorer IQ, based on SABRE score that uses a common methodology for 3D‐EF and 3D‐GLS, and all other LV myocardial indices remained independent of confounders, except for peak longitudinal strain and end‐systolic volume (adjusted absolute standardized bias: ≈2% to 7%, ≈15% to 18%, and ≈4% to 7% for strain, rotational, and volume indices, respectively; Table S6).

There were 90 (17%) participants with 3D‐EF<50%; there was no evidence that low 3D‐EF modified associations between IQ scores and 3D‐EF and 3D‐GLS (Tables S7 and S8), but the number of individuals with abnormal EF was small and the estimates were imprecise.

### Relationships With Frame Rate in SABRE

The acquisition rate was 18.5±3.3 frames/cycle (n=529). The acquisition rate was associated with 3D‐EF and 3D‐GLS and all other global and averaged segmental peak LV strain indices, independent of confounders (Table S9). Conversely, acquisition rate was not associated with LV rotational and volume indices apart from stroke volume (Table S9).

### Effect of Impaired Image Quality in Experimental Studies

Five out of 23 and 3 out of 21 screened individuals were excluded owing to suboptimal echo windows in the poor technique and neoprene studies, respectively. The acquisition rate was was 21±4 and 21±3 frames/cycle, respectively.

In these 2 different experimental and validation models of individuals with experimentally impaired IQ, either by poor technique or use of neoprene, mean differences between individuals with poor versus good IQ ranged from −2.6 (95% CI, −3.2 to −2.0) to −3.2 (95% CI, −3.9 to −2.5) for 3D‐EF and from −1.2 (95% CI, −1.9 to −0.48) to −2.0 (95% CI, −2.8 to −1.2) for absolute 3D‐GLS (Tables 6 and 7). In the neoprene study, underestimation bias in 3D‐EF and 3D‐GLS was proportional to the extent of degradation in IQ (ie, the poorer the IQ the larger the bias; *P* for trend ≤0.0001 for all). Results were similar for other LV strain and rotational indices and for LV volumes (except for end‐systolic volume) in the poor technique study (Table S10). LV volumes and LV strain and rotational indices were underestimated proportional to the extent of degradation in IQ in the neoprene study (Table S11).

**Table 6:** Comparison of 3D‐EF by Image Quality in the Experimental Studies

Poor technique study (n=18)			
	Good	Suboptimal	*P* value
Mean±SD, %	56.9±3.2	54.3±2.6	
Mean Δ (95% CI), % [absolute standardized bias]	Reference	−2.6 (−3.2 to −2.0) [4.6%]	<0.0001
Intraclass correlation coefficient	0.94	0.78	

Neoprene study (n=18)					
	Extent of bias relative to the reference				*P* (trend)
	Reference	Mild	Moderate	Severe	
Mean± SD	55.4±2.5	54.2±2.5	53.1±2.1	52.2±2.1	
Mean Δ (95% CI) [absolute standardized bias]	Reference	−1.2 (−1.9 to −0.46) [2%]	−2.2 (−2.9 to −1.5) [4%]	−3.2 (−3.9 to −2.5) [6%]	<0.0001

3D‐EF indicates 3‐dimensional ejection fraction.

**Table 7:** Comparison of 3D‐GLS by Image‐Quality in the Experimental Studies

Poor technique study (n=18)			
	Good	Suboptimal	*P* value
Mean±SD	21.4±1.9	20.2±2.5	
Mean‐Δ (95% CI), % [absolute standardized bias]	Reference	−1.2 (−1.9 to −0.48) [5.6%]	0.001
Intraclass correlation coefficient	0.62	0.41	

Neoprene study (n=18)					
	Extent of bias relative to the reference				*P* (trend)
	Reference	Mild	Moderate	Severe	
Mean±SD, %	20.8±1.7	20.3±1.6	19.9±1.8	18.7±2.0	
Mean‐Δ (95% CI), % [absolute standardized bias]	Reference	−0.5 (−1.3 to 0.3) [2%]	−0.9 (−1.7 to −0.1) [4%]	−2.0 (−2.8 to −1.2) [10%]	<0.0001

3D‐GLS indicates 3‐dimensional global longitudinal strain.

Reliability from test‐retest was excellent for 3D‐EF and volumes irrespective of IQ, fair to good for LV strain indices when IQ was optimal, but less good for poor quality images, and poor for rotational indices irrespective of IQ (Tables 6 and 7, Table S10, Figure S2). The effect of IQ on test‐retest reproducibility is shown in (Table S12 and Figure S3). Poor quality images showed a higher mean difference and wider limits of agreement for all LV myocardial indices compared with analyses performed using good images. Intraobserver reproducibility based on rereading the same scans showed excellent reproducibility for all LV myocardial indices (Table S13). Interobserver reproducibility was good to excellent for all LV myocardial indices but lower than intraobserver reproducibility especially for rotational indices (Table S14).

## DISCUSSION

3DE is an exciting technology; however, to be useful, it needs to be feasible and to give unbiased and reproducible results.^21^ In a large triethnic population‐based sample of older people, based on IQ, the feasibility of 3DE LV analysis was low (≈41%). This is worse than the feasibility of LV EF by 2D echocardiography observed in this study (61%) and substantially poorer than most conventional echocardiography measures (≈93–95%) as reported previously in SABRE,^12^ but it is slightly better than the feasibility of LV rotation using 2D‐STE (31%) that we have reported previously in the same cohort.^22^ The prevalence of good IQ, defined by the 2015 ASE/EACVI criteria, was 33.6% (178 out of 529) and 71.3% (377 out of 529) for 3D‐EF and 3D‐GLS, respectively in a subset of individuals (n=529) with feasible 3DE images. Even when analysis was feasible, values of LV myocardial indices including 3D‐EF and 3D‐GLS were systematically lower in individuals with poorer ultrasound IQ. These findings (ie, systematic downward bias) were consistent when other graded IQ scoring systems were used and the bias was more marked with poorer IQ. Further, findings from 2 different experimental models confirmed these observations and also showed that the poorer the IQ, the larger the underestimation bias. Poor IQ also impaired the test‐retest reliability/reproducibility of LV myocardial indices, particularly LV strain.

The importance of IQ for 3DE has been discussed previously.^6^, ^7^, ^8^, ^23^ Trache et al.^8^ reported better agreement between 2D‐STE and 3D‐STE LV strains and EF when poor quality segments were excluded.^8^ Kawamura et al.^23^ compared 3D‐EF and volumes with cardiac magnetic resonance and reported greater mean differences and wider limits of agreement with lower 3DE data set IQ score.^23^ Muraru et al.^7^ reported a correlation between IQ and 3D‐STE derived LV strain indices in healthy volunteers. The observational nature of these studies, however, means that confounding by subclinical disease or some other physical characteristic cannot be excluded. We show in a population‐based sample that IQ is associated with biased estimates of LV 3D‐EF and 3D‐GLS and other strain, rotational, and volume indices even after adjusting for multiple confounders. Our work also adds to that of Mor‐Avi et al. who reported a progressively increased bias with decreasing level of operator experience when measuring end‐diastolic and end‐systolic volumes by real‐time 3DE.^24^ Temporal resolution is another influence on 3D‐STE‐derived strain indices.^5^, ^7^ Our findings agree with earlier studies,^5^, ^7^ which showed reduced 3D strain values with lower frames/cycle.

We found a similar reliability of LV myocardial indices by means of transthoracic 3DE to previous studies using optimal images.^7^, ^25^, ^26^, ^27^ Poor quality images modestly impaired reproducibility of volume indices, whereas the reproducibility of strain indices was more affected. The reproducibility of rotational indices was poor irrespective of IQ.

Our feasibility of 3DE is similar to that achieved in another multiethnic population‐based study (ARIC [Atherosclerosis Risk in Communities], 36.4%),^27^ but lower than reported in some healthy^7^, ^26^, ^28^, ^29^ or selected samples.^30^, ^31^ Unlike ARIC, which reported no differences in demographics and clinical characteristics between included and excluded subjects, we found that participants in whom 3DE LV analysis could not be performed were older, heavier, and more likely to be of South Asian ethnicity and to have hypertension, diabetes, and a history of coronary heart disease. The reason for these associations with feasibility requires further investigation but could relate to differences in body morphology or fat distribution.

This study has limitations. SABRE is a UK‐based triethnic study of older individuals and our observations may not generalize to other populations. Although SABRE is a population‐based study, it should not be regarded as free of bias as people who agree to participate in studies may differ from those who do not and exclusion of individuals with unanalyzable images potentially introduces large, albeit unavoidable, selection bias. In the experimental studies, 2 approaches were used to impair IQ; these may not replicate pathophysiological conditions influencing IQ (eg, emphysema or surgical scar). The ultrasound machines and transducers differed between the observational and experimental studies; this may limit the extrapolation of findings between studies, although it is notable that the estimates of magnitude of bias due to IQ are very similar. We did not test our approach using software from different vendors. IQ‐related bias could vary between different software; however, a previous study reported that IQ only made a minor contribution to differences between software from different vendors.^6^

## CONCLUSIONS

The findings of this large study indicate that 3DE LV analyses, including 3D‐EF and 3D‐GLS, had low feasibility and that feasible but poorer quality images gave systematically lower values of EF and deformation. This has the potential to be an important neglected source of bias, because the size of the IQ‐related bias is similar to the associations reported in disease.^32^, ^33^, ^34^ Hence, although EF and GLS by means of transthoracic 3DE have potential advantages over 2D echocardiography, further technical development may be required to improve the utility of 3DE in clinical practice.

## Sources of Funding

L.A.S. is supported by a scholarship grant from Imam Abdulrahman Bin Faisal University for her postgraduate studies (PhD) at University College London. A.D.H. and N.C. work in a unit that receives support from the UK Medical Research Council (MRC) (Programme Code MC_UU_12019/1), the British Heart Foundation (BHF) (PG/15/75/31748, CS/15/6/31468, CS/13/1/30327, SPG 2822621), and the National Institute for Health Research UCL Hospitals Biomedical Research Centre. Park received support from BHF (CS/15/6/31468). The Southall study was supported by the UK MRC, the British Diabetic Association (now Diabetes UK), the Wellcome Trust, and the BHF. The Brent study was supported by the UK MRC. The SABRE study was supported by the Wellcome Trust (082464) and BHF (SP/07/001/23603, CS/13/1/30327). The SABRE 3D Heart substudy was funded by the BHF (PG/08/103/26133).

## Disclosures

None.

**Figure:**
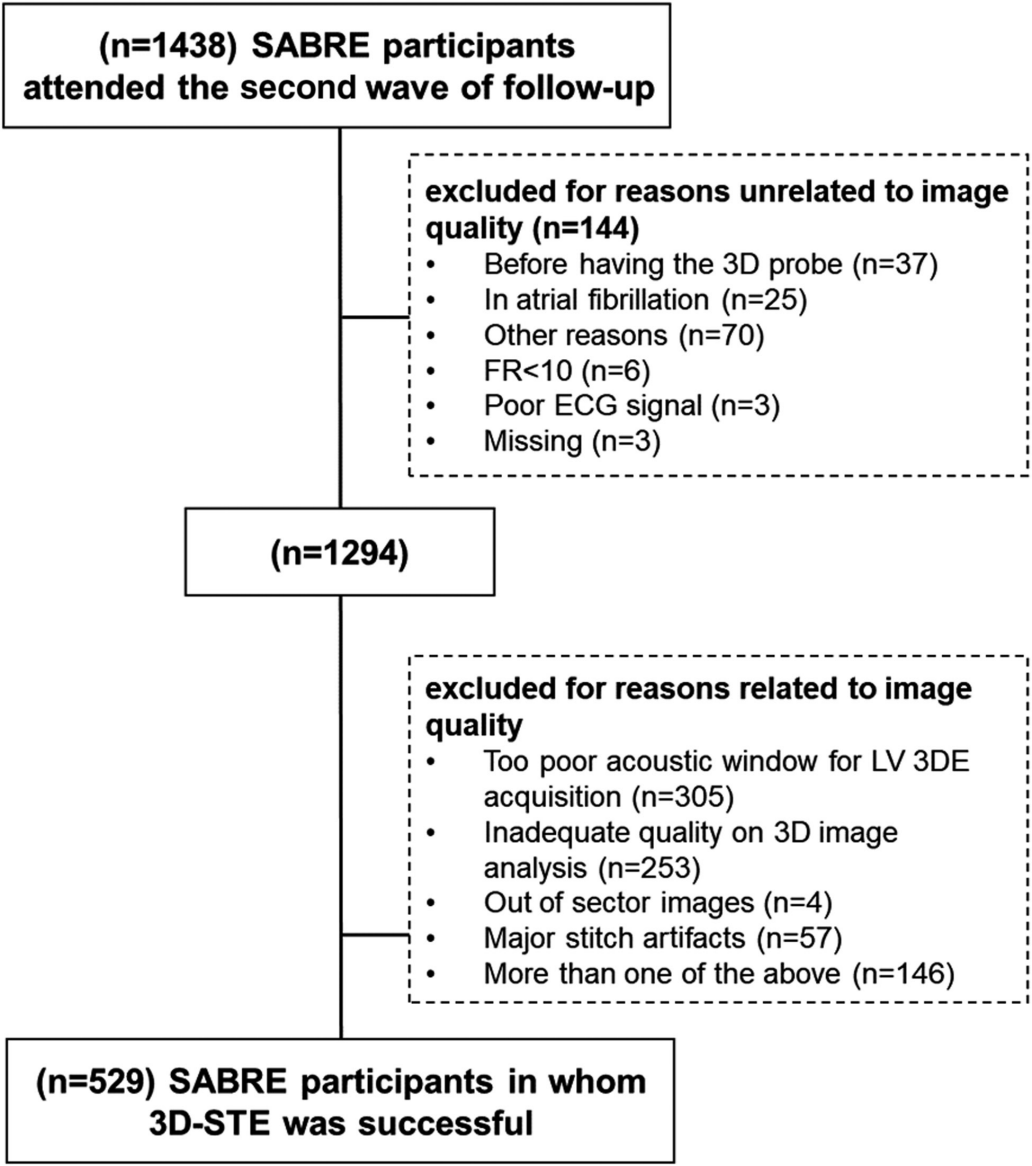
Flow chart showing the enrollment of SABRE participants in the present study. 3DE indicates 3‐dimensional echocardiography; 3D‐STE, 3‐dimensional speckle tracking echocardiography; FR, frame rate; LV, left ventricular; and SABRE, Southall and Brent Revisited study.

## Supporting information

Data S1
Tables S1–S14
Figures S1–S3
