# Supplementary material for: Relationship Between Image Quality and Bias in 3D Echocardiographic Measures: Data From the SABRE (Southall and Brent Revisited) Study
Source: J Am Heart Assoc. 2022 Apr 27;11(9):e019183. doi: 10.1161/JAHA.120.019183 (PMC9238620; doi:10.1161/JAHA.120.019183)
Supplement: Supplementary file 1 — Data S1 Tables S1–S14 Figures S1–S3 [file JAH3-11-e019183-s001.pdf]

Supplemental Material

Data S1.

Supplemental Methods

Image analysis

Images were analysed using 4D LV-Analysis© software (TomTec Imaging Systems GmbH, Germany, 2015) by a single experienced reader. For the experimental studies, analysis of 3DE LV datasets was performed in all datasets obtained per participant (i.e. 4 analyses/participant). For the observational study, the analysis was performed according to a pre-specified protocol, and image quality was defined as follows:

1) **Good(score-1)**=clear visualization of endocardium in all 16 segments in both ED and ES frames.
2) **Fair(score-2)=**unclear visualization of endocardium in ≤2 segments or presence of minor artefacts e.g. apical noise.
3) **Adequate(score-3)**=unclear visualization of endocardium in ≤6 segments.
4) **Poor(score-4)**=unclear visualization of endocardium in >6 segments in ED or ES frames, but the endocardium can still be tracked with confidence throughout the cardiac-cycle using the adjacent segments as a reference.
5) Unacceptable image quality was defined as presence of major stitching artefacts preventing reliable tracking of the endocardium, unacceptable visualization of the LV endocardial boundaries, or ≥4 segments of the LV wall being outside of the image sector.

The software automatically selected and displayed three standard apical views and one short-axis view. Alignment of the longitudinal axis of the LV in all apical views were further modified manually if needed using two anatomical landmarks at both ends (the mitral valve annulus and the apex). The endocardial borders were then defined automatically by the software in all apical views at end-diastole. Manual adjustments could be made but these were kept as minimal as possible to enhance reproducibility. The software then tracked the endocardium throughout the cardiac cycle in 3D space from which the 3D LV endocardial shell was constructed. The tracing of LV endocardial boundaries was further adjusted manually when needed in ED and ES frames. The software then divided the LV into 16 segments and generated curves and maps of global and segmental volumetric and deformation indices.

Table S1. Feasibility of 2D-guided M-mode LV linear dimensions* in 1438 SABRE participants.

LVIDd	1354(94%)
LVIDs	1352(94%)
IVSd	1354(94%)
IVSs	1352(94%)
PWd	1354(94%)
PWs	1353(94%)

*LV volumes from conventional 2D-echocardiography were calculated by the Teichholz formula using the linear dimensions from which 2D LV ejection fraction was derived.

IVSd, diastolic interventricular septal thickness; IVSs, systolic interventricular septal thickness; LV, left ventricle; LVIDd, diastolic left ventricular internal diameter; LVIDs, systolic left ventricular internal diameter; PWTd, diastolic posterior wall thickness; PWTs, systolic posterior wall thickness.

Table S2. Baseline characteristics of SABRE participants with and without 3DE LV analysis.

	**+ TomTec 3DE LV analysis (n=529)**	**- TomTec 3DE LV analysis (n=878)**	**P value**
Age, y	69.1±6.1	70.0±6.1	0.009
Male, n(%)	405(76.6)	664(75.6)	0.69
Ethnicity, European/South Asian/African Caribbean(%)	51.6/28.5/20.0	45.1/40.9/14.0	<0.0001
Systolic blood pressure, mmHg	140.2±17.9	140.1±17.8	0.96
Diastolic blood pressure, mmHg	76.5±9.6	77.3±9.8	0.14
Heart rate	67.2±11.4	68.9±12.7	0.008
Body mass index, kg/m^2	26.1±3.5	28.5±5.2	<0.0001
Waist: hip ratio	0.96±0.07	0.99±0.08	<0.0001
Hypertension, n(%)	301(56.9)	642(73.1)	<0.0001
Known diabetes, n(%)	118(22.3)	322(36.7)	<0.0001
Prior coronary heart diseases, n(%)	89(16.8)	266(30.3)	<0.0001
Smoking status, never/ex/current(%)	54.1/38.1/7.8	58.7/36.0/5.3	0.09

Data are mean±SD or n(%).

Table S3. Relationships with image quality for 3D-EF and 3D-GLS in the SABRE study(n=529).

**2015 ASE/EACVI guidelines-based image-quality score**			
**coefficient(95% CI), *p***			
	**Unadjusted**	**Adjusted**	**Absolute standardized bias (%)***
**3D-EF, %**	-2.9(-3.9, -1.8), <0.0001	-2.5(-3.6, -1.5), <0.0001	4.6%
**3D-GLS, %**	-0.6(-1.1, 0.0), 0.058	-0.7(-1.2, -0.1), 0.018	3.7%
**Poor image-quality segments score**			
**coefficient(95% CI), *p* (per 1-point increment in score)**			
	**Unadjusted**	**Adjusted**	**Absolute standardized bias (%)**
**3D-EF, %**	-1.3(-1.8, -0.8), <0.0001	-1.2(-1.7, -0.7), <0.0001	2.2%
**3D-GLS, %**	-0.5(-0.8, -0.2), <0.0001	-0.5(-0.7, -0.2), <0.0001	2.6%
**SABRE image-quality score**			
**coefficient(95% CI), *p* (per 1-point increment in score)**			
	**Unadjusted**	**Adjusted**	**Absolute standardized bias (%)**
**3D-EF, %**	-2.1(-2.8, -1.3), <0.0001	-2.0(-2.7, -1.3), <0.0001	3.7%
**3D-GLS, %**	-0.4(-0.8, -0.1), 0.025	-0.4(-0.8, -0.0), 0.030	2.1%

Coefficients are unstandardized coefficients of regression. Adjustment was performed for age, sex, ethnicity, height, weight, heart rate, history of percutaneous coronary intervention and/or coronary artery bypass graft and/or history of chronic obstructive pulmonary disease. *The extent of adjusted bias represented in standardized terms relative to the overall mean. Abbreviations: CI, confidence interval; EF, ejection fraction; and GLS, global longitudinal strain.

Table S4. 3DE derived LV myocardial indices by poor image-quality segments score in the SABRE study(n=529).

	None-segment	1-segment	2-sgements	≥3-segments	P value
**n(%)**	**63(11.9)**	**115(21.7)**	**219(41.4)**	**132(25.0)**	
**EDV, ml/m^2**					
Mean±SD	58.4±11.8	58.6±14.4	57.9±13.9	54.7±11.4	0.067
Mean Δ(95% CI)	Reference	0.2(-3.9, 4.2)	-0.5(-4.2, 3.2)	-3.7(-7.7, 0.2)	
**ESV, ml/m^2**					
Mean±SD	25.7±7.1	26.4±8.4	27.6±9.3	25.9±7.1	0.179
Mean Δ(95% CI)	Reference	0.7(-1.9, 3.3)	1.9(-0.4, 4.3)	0.2(-2.3, 2.7)	
**SV, ml**					
Mean±SD	57.7±13.2	59.7±15.6	56.1±13.7	53.7±13.2	0.009
Mean Δ(95% CI)	Reference	2.0(-2.3, 6.3)	-1.6(-5.5, 2.3)	-3.9(-8.1, 0.3)	
**n(%)**	**63(11.9)**	**103(19.5)**	**212(40.1)**	**151(28.5)**	
**GCS, %**					
Mean±SD	27.7±3.3	26.8±4.4	25.2±4.0	24.5±3.8	<0.0001
Mean Δ(95% CI)	Reference	-1.0(-2.2, 0.3)	-2.5(-3.6, -1.4)	-3.2(-4.3, -2.0)	
**Peak averaged CS, %**					
Mean±SD	27.9±3.3	27.0±4.6	25.4±4.0	24.6±3.7	<0.0001
Mean Δ(95% CI)	Reference	-0.9(-2.2, 0.3)	-2.6(-3.7, -1.5)	-3.4(-4.5, -2.2)	
**Peak averaged LS, %**					
Mean±SD	19.1±2.7	18.9±2.8	18.0±2.9	18.1±3.2	0.008
Mean Δ(95% CI)	Reference	-0.2(-1.1, 0.7)	-1.1(-1.9, -0.2)	-1.0(-1.9, -0.2)	
**Peak averaged PTS, %**					
Mean±SD)	32.8±3.4	32.3± 4.4	30.8±4.0	30.2±3.9	<0.0001
Mean Δ(95% CI)	Reference	-0.5(-1.8, 0.7)	-2.0(-3.1, -0.9)	-2.6(-3.8, -1.5)	
**Peak averaged RS, %**					
Mean±SD	39.4±4.4	38.5±5.5	36.4±4.9	35.8±5.1	<0.0001
Mean Δ(95% CI)	Reference	-0.9(-2.5, 0.7)	-3.0(-4.4, -1.6)	-3.6(-5.1, -2.1)	
**Peak basal rotation, °**					
Mean±SD	6.2±3.4	6.0±3.3	5.3± 3.2	5.0±3.3	0.014
Mean Δ(95% CI)	Reference	-0.2(-1.2, 0.9)	-0.9(-1.8, 0.01)	-1.2(-2.2, -0.3)	
**Peak apical rotation, °**					
Mean±SD	9.4±4.2	9.5±4.3	8.0±4.3	7.3±4.5	0.0002
Mean Δ(95% CI)	Reference	0.1(-1.3, 1.4)	-1.4(-2.6, -0.2)	-2.1(-3.4, -0.8)	
**Peak twist, °**					
Mean±SD	15.2±6.9	15.2±6.9	13.0±6.5	11.8±7.1	0.0001
ean Δ(95% CI)	Reference	-0.0(-2.1, 2.1)	-2.2(-4.1, -0.3)	-3.5(-5.5, -1.5)	
**Peak torsion, °/cm**					
Mean±SD	1.9±0.9	1.9±0.9	1.6±0.8	1.5±0.9	0.0001
Mean Δ(95% CI)	Reference	-0.0(-0.3, 0.2)	-0.3(-0.6, -0.1)	-0.5(-0.7, -0.2)	

Abbreviations: CS, circumferential strain; CI, confidence interval; EDV, end-diastolic volume; ESV, end-systolic volume; GCS, global circumferential strain; LS, longitudinal strain; LV, left ventricular; PTS, principle tangential strain; RS, radial strain; SD, standard deviation; and SV, stroke volume.

Table S5. 3DE derived LV myocardial indices by SABRE image-quality score in the SABRE study(n=529).

	Good	Fair	Adequate	Poor	P value
**n(%)**	**19(3.6)**	**235(44.4)**	**239(45.2)**	**36(6.8)**	
**EDV, ml/m²**					
Mean±SD	58.1±10.0	58.5±13.3	56.4±13.9	55.3±9.5	0.260
Mean Δ(95% CI)	Reference	0.4(-5.8, 6.6)	-1.7(-7.9, 4.5)	-2.8(-10.2, 4.5)	
**ESV, ml/m²**					
Mean±SD	25.1[21.4-27.7]*	25.2[21.6-29.9]*	26.1[21.9-29.8]*	25.8[21.7-30.0]*	0.808#
Mean Δ(95% CI)	Reference	0.9(-3.0, 4.8)	1.8(-2.2, 5.7)	0.8(-3.9, 5.5)	
**SV, ml**					
Mean±SD	57.0±11.9	59.1±14.9	54.2±13.2	54.5±12.4	0.002
Mean Δ(95% CI)	Reference	2.0(-4.5, 8.5)	-2.9(-9.4, 3.7)	-2.5(-10.2, 5.2)	
**GCS, %**					
Mean±SD	27.9±3.2	26.7±4.1	24.5±3.9	24.3±3.1	<0.0001
Mean Δ(95% CI)	Reference	-1.2(-3.0, 0.7)	-3.3(-5.2, -1.5)	-3.6(-5.8, -1.4)	
**Peak averaged CS, %**					
Mean±SD	28.2±3.2	26.9±4.1	24.7±3.9	24.2±3.1	<0.0001
Mean Δ(95% CI)	Reference	-1.3(-3.1, 0.6)	-3.5(-5.3, 1.7)	-3.9(-6.1, -1.8)	
**Peak averaged LS, %**					
Mean±SD	19.1±2.0	18.6±2.7	17.9±3.1	19.2±3.3	0.005
Mean Δ(95% CI)	Reference	-0.5(-1.9, 0.9)	-1.3(-2.6, -0.1)	0.1(-1.6, 1.7)	
**Peak averaged PTS, %**					
Mean±SD	33.0±3.3	32.0±4.1	30.2±4.0	30.5±3.2	<0.0001
Mean Δ(95% CI)	Reference	-1.0(-2.9, 0.8)	-2.8(-4.7, -0.9)	-2.5(-4.7, -0.3)	
**Peak averaged RS, %**					
Mean±SD	39.7±3.9	38.2±5.1	35.7±5.2	36.5±4.6	<0.0001
Mean Δ(95% CI)	Reference	-1.5(-3.8, 0.9)	-4.0(-6.3, -1.6)	-3.2(-6.0, -0.4)	
**Peak basal rotation, °**					
Mean±SD	7.8±2.8	5.7±3.3	5.1± 3.1	4.4±3.6	0.0007
Mean Δ(95% CI)	Reference	-2.0(-3.5, -0.5)	-2.6(-4.1, -1.1)	-3.3(-5.1, -1.5)	
**Peak apical rotation, °**					
Mean±SD	10.2[8.6-11.9]*	8.5[5.7-11.7]*	7.3[4.6-10.4]*	6.5[3.3-9.8]*	0.0001#
Mean Δ(95% CI)	Reference	-1.4(-3.4, 0.7)	-2.7(-4.7, -0.6)	-3.6(-6.0, -1.1)	
**Peak twist, °**					
Mean±SD	17.8±6.3	14.4±6.8	12.4±6.7	10.7±7.4	<0.0001
Mean Δ(95% CI)	Reference	-3.4(-6.6, -0.2)	-5.4(-8.6, -2.2)	-7.1(-10.9, -3.3)	
**Peak torsion, °/cm**					
Mean±SD	2.2±0.8	1.8±0.9	1.5±0.8	1.3±1.0	<0.0001
Mean Δ(95% CI)	Reference	-0.4(-0.8, -0.0)	-0.7(-1.1, -0.3)	-0.9(-1.4, -0.4)	

Abbreviations: CS, circumferential strain; CI, confidence interval; EDV, end-diastolic volume; ESV, end-systolic volume; GCS, global circumferential strain; LS, longitudinal strain; LV, left ventricular; PTS, principle tangential strain; RS, radial strain; SD, standard deviation; and SV, stroke volume. # by Kruskal-Wallis. *Data are median[interquartile range].

Table S6. Relationships with image quality for other LV myocardial indcies using SABRE image-quality score in the SABRE study(n=529).

	SABRE image-quality score		
	coefficient(95% CI), *p* (per 1-point increment in score)		
	**Unadjusted**	**Adjusted**	**Absolute standardized bias (%)***
**LV volumetric indices**			
EDV, ml	-1.3(-4.8, 2.2), 0.464	-3.6(-6.6, -0.5), 0.021	3.4%
ESV, ml	1.6(-0.6, 3.7), 0.144	0.5(-1.4, 2.4), 0.622	1.0%
SV, ml	-2.9(-4.7, -1.1), 0.001	-4.0(-5.6, -2.5), <0.0001	7.1%
**LV strain indices**			
GCS, %	-1.6(-2.1, -1.1), <0.0001	-1.6(-2.1, -1.1), <0.0001	6.3%
Peak averaged LS, %	-0.3(-0.7, 0.1), 0.145	-0.3(-0.6, 0.1), 0.175	1.6%
Peak averaged CS, %	-1.7(-2.2, -1.2), <0.0001	-1.7(-2.1, -1.2), <0.0001	6.6%
Peak averaged RS, %	-1.6(-2.3, -1.0), <0.0001	-1.6(-2.2, -1.0), <0.0001	4.3%
Peak averaged PTS, %	-1.2(-1.7, -0.7), <0.0001	-1.2(-1.7, -0.7), <0.0001	3.9%
**LV rotational indices**			
Peak basal rotation, °	-0.8(-1.2, -0.4), <0.0001	-0.8(-1.3, -0.4), <0.0001	14.8%
Peak apical rotation, °	-1.2(-1.8, -0.7), <0.0001	-1.2(-1.8, -0.7), <0.0001	14.5%
Peak twist, °	-2.1(-3.0, -1.3), <0.0001	-2.2(-3.1, -1.3), <0.0001	16.4%
Peak torsion, °/cm	-0.3(-0.4, -0.2), <0.0001	-0.3(-0.4, -0.2), <0.0001	17.6%

Coefficients are unstandardized coefficients of regression. Adjustment was performed for age, sex, ethnicity, height, weight, heart rate, history of percutaneous coronary intervention and/or coronary artery bypass graft and/or history of chronic obstructive pulmonary disease. *The extent of adjusted bias represented in standardized terms relative to the overall mean. These results are shown for SABRE image-quality score only as other definitions of image quality differ between EF and GLS. Abbreviations: CS, circumferential strain; CI, confidence interval; EDV, end-diastolic volume; ESV, end-systolic volume; GCS, global circumferential strain; LS, longitudinal strain; LV, left ventricular; PTS, principle tangential strain; RS, radial strain; and SV, stroke volume.

Table S7. Relationships between image quality and 3D-EF and 3D-GLS according to 3D-EF in the SABRE study(n=529).

	≥50% EF (n=439)		<50% EF (n=90)	
**2015 ASE/EACVI guidelines-based image-quality score**				
**coefficient(95% CI), *p***				
	**Unadjusted**	**Adjusted**	**Unadjusted**	**Adjusted**
**3D-EF, %**	-1.4(-2.2, -0.6), 0.001	-1.1(-1.9, -0.3), 0.005	2.4(-0.8, 5.6), 0.141	3.2(-0.3, 6.7), 0.076
**3D-GLS, %**	-0.2(-0.8, 0.3), 0.409	-0.3(-0.8, 0.3), 0.360	0.3(-1.0, 1.6), 0.640	0.1(-1.2, 1.4), 0.897
**Poor image-quality segments score**				
**coefficient(95% CI), *p* (per 1-point increment in score)**				
	**Unadjusted**	**Adjusted**	**Unadjusted**	**Adjusted**
**3D-EF, %**	-0.7(-1.1, -0.3), 0.001	-0.5(-0.9, -0.1), 0.009	1.1(-0.3, 2.6), 0.124	1.2(-0.3, 2.8), 0.125
**3D-GLS, %**	-0.3(-0.5, 0.0), 0.034	-0.2(-0.5, 0.0), 0.079	0.2(-0.6, 1.1), 0.604	-0.03(-0.9, 0.9), 0.942
**SABRE image-quality score**				
**coefficient(95% CI), *p* (per 1-point increment in score)**				
	**Unadjusted**	**Adjusted**	**Unadjusted**	**Adjusted**
**3D-EF, %**	-1.2(-1.8, -0.7), <0.0001	-1.1(-1.7, -0.6), <0.0001	0.3(-1.5, 2.1), 0.738	0.1(-1.9, 2.0), 0.945
**3D-GLS, %**	-0.1(-0.5, 0.2), 0.424	-0.1(-0.5, 0.3), 0.564	0.4(-0.6, 1.5), 0.412	0.1(-1.0, 1.2), 0.805

Coefficients are unstandardized coefficients of regression. Adjustment was performed for age, sex, ethnicity, height, weight, heart rate, history of percutaneous coronary intervention and/or coronary artery bypass graft and/or history of chronic obstructive pulmonary disease. Abbreviations: CI, confidence interval; EF, ejection fraction; and GLS, global longitudinal strain.

Table S8. 3D-EF and 3D-GLS by image quality scores according to 3D-EF in the SABRE study(n=529).

**2015 ASE/EACVI guidelines-based image-quality score**					
	**Good**	**Poor**			**P value**
**3D-EF, %**	Mean±SD				
EF≥50%	(n=167)	(n=272)			
	56.5±4.5	55.1±3.8			0.0006
EF<50%	(n=11)	(n=79)			
	42.4±4.1	44.8±5.2			0.141
**3D-GLS%**	Mean±SD				
EF≥50%	(n=325)	(n=114)			
	19.7±2.5	19.5±2.9			0.409
EF<50%	(n=52)	(n=38)			
	15.7±3.1	16.0±2.8			0.639
**Poor image-quality segments score**					
	**None-segment**	**1-segment**	**2-segments**	**≥3-segments**	**P value**
**3D-EF, %**			Mean±SD		
EF≥50%	(n=60)	(n=107)	(n=171)	(n=101)	
	57.0±4.1	56.3±4.8	55.1±3.9	55.1±3.6	0.004
EF<50%	(n=3)	(n=8)	(n=48)	(n=31)	
	44.4*	41.6±4.6	44.3±5.6	45.5±4.5	0.285
**3D-GLS%**			Mean±SD		
EF≥50%	(n=60)	(n=96)	(n=170)	(n=113)	
	20.1±2.7	20.1± 2.3	19.4±2.5	19.5±2.9	0.009
EF<50%	(n=3)	(n=7)	(n=42)	(n=38)	
	17.2*	14.1±4.0	15.9±3.0	16.0±2.8	0.389
**SABRE image-quality score**					
	**Good**	**Fair**	**Adequate**	**Poor**	**P value**
**3D-EF, %**		Mean±SD			
EF≥50%	(n=18)	(n=212)	(n=181)	(n=28)	
	57.2±3.6	56.4±4.5	54.7±3.7	54.7±3.2	0.0001
EF<50%	(n=1)	(m=23)	(n=58)	(n=8)	
	43.1*	44.3±4.2	44.5±5.6	44.9±3.7	0.985
**3D-GLS%**		Mean±SD			
EF≥50%	(n=18)	(n=212)	(n=181)	(n=28)	
	20.1±2.0	19.8±2.4	19.3±2.8)	20.4±2.9	0.093
EF<50%	(n=1)	(m=23)	(n=58)	(n=8)	
	17.3*	15.4±3.1	15.9±2.9	16.6±3.1	0.722

*Standard deviation has not been presented for data where n ≤ 3, only the mean value is shown. Abbreviations: EF, ejection fraction; GLS, global longitudinal strain; and SD, standard deviation.

Table S9. Relationships between frames per cycle and 3DE derive LV myocardial indcies in the SABRE study(n=529).

	coefficient(95% CI), *p* (per frames/cycle)	
	**Unadjusted**	**Adjusted**
**LV volumetric indices**		
3D-EF, %	0.4(0.2, 0.5), <0.0001	0.4(0.2, 0.6), 0.001
EDV, ml	0.6(-0.1, 1.3), 0.117	0.5(-0.4, 1.4), 0.277
ESV, ml	-0.2(-0.7, 0.2), 0.336	-0.2(-0.8, 0.4), 0.436
SV, ml	0.8(0.4, 1.1), <0.0001	0.7(0.3, 1.2), 0.003
**LV strain indices**		
3D-GLS, %	0.1(0.0, 0.2), 0.012	0.1(0.0, 0.2), 0.029
GCS, %	0.2(0.1, 0.3), <0.0001	0.2(0.1, 0.4), 0.001
Peak averaged LS, %	0.1(0.0, 0.2), 0.021	0.1(0.0, 0.2), 0.051
Peak averaged CS, %	0.2(0.1, 0.3), <0.0001	0.2(0.1, 0.4), 0.002
Peak averaged RS, %	0.2(0.1, 0.4), 0.001	0.3(0.1, 0.5), 0.003
Peak averaged PTS, %	0.1(0.0, 0.2), 0.010	0.2(0.1, 0.4), 0.010
**LV rotational indices**		
Peak basal rotation, °	0.0(-0.1, 0.1), 0.509	0.0(-0.1, 0.2), 0.762
Peak apical rotation, °	0.0(-0.1, 0.1), 0.913	0.1(-0.1, 0.3), 0.193
Peak twist, °	0.0(-0.2, 0.1), 0.664	0.1(-0.1, 0.4), 0.298
Peak torsion, °/cm	0.0(-0.0, 0.0), 0.876	0.0(-0.0, 0.1), 0.204

Coefficients are unstandardized coefficients of regression. Adjustment was performed for age, sex, ethnicity, height, weight, heart rate, history of percutaneous coronary intervention and/or coronary artery bypass graft and/or history of chronic obstructive pulmonary disease. Abbreviations: CS, circumferential strain; CI, confidence interval; EDV, end-diastolic volume; EF, ejection fraction; ESV, end-systolic volume; GCS, global circumferential strain; GLS, global longitudinal strain; LS, longitudinal strain; LV, left ventricular; PTS, principle tangential strain; RS, radial strain; and SV, stroke volume.

Table S10. Comparison of 3DE derived LV myocardial indices by image quality in the (experimental) poor technique study).

	Mean±SD		Bias		ICC	
	Good	Sub-optimal	Mean-Δ(95% CI)	Absolute standardized bias (%)*	Good*	Sub-optimal†
**LV volumetric indices**						
EDV, ml	123.9±19.7	117.7±18.6	-6.2(-8.7, -3.7)	5.0%	0.97	0.91
ESV, ml	53.6±11.0	53.9±9.6	0.2(-1.1, 1.5)	0.4%	0.96	0.89
SV, ml	70.3±9.9	63.9±10.0	-6.4(-8.0, -4.8)	9.1%	0.97	0.90
**LV strain indices**						
GCS, %	28.1±2.6	25.7±2.1	-2.3(-2.9, -1.8)	8.2%	0.88	0.52
Peak averaged CS, %	28.3±2.6	25.7±2.5	-2.6(-3.2, -2.0)	9.2%	0.86	0.54
Peak averaged LS, %	20.4±2.0	19.7±2.4	-0.7(-1.4, -0.02)	3.4%	0.66	0.39
Peak averaged PTS, %	33.1±2.6	31.4±2.8	-1.6(-2.4, -0.9)	4.8%	0.82	0.36
Peak averaged RS, %	40.9±3.0	38.2±3.0	-2.7(-3.4, -2.0)	6.6%	0.80	0.51
**LV rotational indices**						
Peak basal rotation, °	8.0±3.9	6.7±5.1	-1.3(-2.7, 0.2)	16.3%	0.54	0.60
Peak apical rotation, °	6.3±2.7	4.6±3.0	-1.6(-2.7, -0.6)	25.4	0.41	0.24
Peak twist, °	13.7±5.8	10.8±7.0	-2.9(-5.2, -0.6)	21.2%	0.40	0.45
Peak torsion, °/cm	1.6±0.7	1.2±0.8	-0.3(-0.6, -0.06)	18.8	0.37	0.40

Abbreviations: CS, circumferential strain; CI, confidence interval; EDV, end-diastolic volume; ESV, end-systolic volume; GCS, global circumferential strain; ICC, intraclass correlation coefficient; LS, longitudinal strain; LV, left ventricular; PTS, principle tangential strain; RS, radial strain; and SV, stroke volume. *ICC based on un-degraded images. †ICC based on degraded images.

Table S11. The extent of bias proportional to the impairment in image quality of 3DE derived LV myocardial indices in the (experimental) neoprene study.

		Extent of bias relative to the reference			P (trend)
	Reference	Mild	Moderate	Severe	
**EDV, ml**					
Mean Δ(95% CI)	-	-7.8(-14.5, -1.0)	-11.8(-18.5, -5.0)	-19.5(-26.3, -12.8)	<0.0001
*Absolute standardized bias (%)**	-	5.6%	8.5%	14%	
Mean±SD	139.6±25.5	131.8±20.9	127.8±19.9	120.0±22.3	
**ESV, ml**					
Mean Δ(95% CI)	-	-1.9(-5.1, 1.3)	-2.5(-5.7, 0.69)	-5.1(-8.3, -1.8)	0.002
*Absolute standardized bias (%)**	-	3.0%	4.0%	8.2%	
Mean± SD	62.5±13.1	60.6±11.6	59.9±10.5	57.4±11.1	
**SV, ml**					
Mean Δ(95% CI)	-	-5.8(-9.6, -2.1)	-9.3(-13.0, -5.5)	-14.5(-18.3, -10.7)	<0.0001
*Absolute standardized bias (%)**	-	7.5%	12.1%	18.8%	
Mean±SD	77.1±13.1	71.2±10.1	67.8±10.0	62.6±11.6	
**GCS, %**					
Mean Δ(95% CI)	-	-1.6(-2.4, 0.9)	-1.8(-2.5, -1.0)	-2.6(-3.4, -1.9)	<0.0001
*Absolute standardized bias (%)**	-	6.1%	6.9%	9.9%	
Mean±SD	26.2±2.2	24.6±1.9	24.5±1.3	23.6±2.1	
**Peak averaged CS, %**					
Mean Δ(95% CI)	-	-1.4(-2.3, -0.5)	-1.6(-2.5, -0.7)	-2.9(-3.8, -2.0)	<0.0001
*Absolute standardized bias (%)**	-	5.4%	6.2%	11.2%	
Mean±SD	26.0±2.0	24.6±2.0	24.5±1.6	23.1±2.5	
**Peak averaged LS,** %					
Mean Δ(95% CI)	-	-0.6(-1.5, 0.3)	-1.1(-2.0, -0.2)	-2.0(-2.9, -1.1)	<0.0001
*Absolute standardized bias (%)**	-	2.9%	5.4%	9.8%	
Mean±SD	20.5±1.6	19.9±1.5	19.4±1.9	18.5±2.2	
**Peak averaged PTS, %**					
Mean Δ(95% CI)	-	-1.0(-2.0, 0.0)	-1.4(-2.4, -0.4)	-1.8(-2.8, -0.8)	<0.0001
*Absolute standardized bias (%)**	-	3.1%	4.4%	5.7%	
Mean±SD	-31.8±1.8	30.7±2.0	30.4±1.7	29.9±2.3	
**Peak averaged RS, %**					
Mean Δ(95% CI)	-	-1.7(-2.6, -0.7)	-2.2(-3.1, -1.3)	-4.0(-5.0, -3.1)	<0.0001
*Absolute standardized bias (%)**	-	4.3%	5.6%	10.2%	
Mean±SD	39.2±2.2	37.5±2.2	36.9±2.0	35.1±2.5	
**Peak basal rotation, °**					
Mean Δ(95% CI)	-	-0.1(-1.8, 1.5)	-1.9(-3.5, -0.16)	-2.6(-4.3, -0.9)	0.001
*Absolute standardized bias (%)**	-	1.4%	26.8%	36.6%	
Mean±SD	7.1±3.7	6.9±2.8	5.2±2.1	4.5±3.4	
**Peak apical rotation, °**					
Mean Δ(95% CI)	-	-1.1(-2.8, 0.5)	-2.6(-4.3, -1.0)	-3.0(-4.7, -1.4)	<0.0001
*Absolute standardized bias (%)**	-	16.4%	38.8%	44.8%	
Mean±SD	6.7±5.3	5.5±2.1	4.0±2.3	3.6±2.3	
**Peak twist, °**					
Mean Δ(95% CI)	-	-1.5(-4.6, 1.6)	-4.7(-7.8, -1.6)	-5.9(-9.1, -2.8)	<0.0001

*Absolute standardized bias (%)**	-	11.1%	34.8%	43.7%	
Mean±SD	13.5±8.5	12.1±4.7	8.8±4.0	7.6±5.4	
**Torsion,°/cm**					
Mean Δ(95% CI)	-	-0.1(-0.5, 0.2)	-0.5(-0.8, -0.1)	-0.6(-1.0, -0.3)	<0.0001
*Absolute standardized bias (%)**	-	6.7%	33.3%	40%	
Mean±SD	1.5±0.9	1.3±0.5	1.0±0.4	0.8±0.6	

Abbreviations: CS, circumferential strain; CI, confidence interval; EDV, end-diastolic volume; ESV, end-systolic volume; GCS, global circumferential strain; LS, longitudinal strain; LV, left ventricular; PTS, principle tangential strain; RS, radial strain; SD, standard deviation; and SV, stroke volume.

Table S12. Bland & Altman Analysis of 3DE derived LV myocardial indices by image quality (Experimental: poor technique study).

	Good 1 vs. Good 2		Good 1	Good 2	Sub-optimal 1 vs. Sub-optimal 2		Sub-optimal 1	Sub-optimal 2
	**$Mean_{Diff}$ ± SD**	**95% LOA**	**Mean**±SD		**$Mean_{Diff}$ ± SD**	**95% LOA**	**Mean**±SD	
**LV volumetric indices**								
3D-EF, %	0.7±0.8	-0.8, 2.2	57.3±3.3	56.6±3.2	0.2±1.7	-3.2, 3.5	54.4±2.7	54.2±2.6
EDV, ml	-1.4±4.1	-9.5, 6.7	123.2±19.5	124.6±20.5	-0.5±7.9	-16.0, 15.1	117.5±18.4	118.0±19.2
ESV, ml	-1.5±2.3	-6.0, 3.0	52.9±10.9	54.4±11.4	-0.3±4.3	-8.7, 8.2	53.7±9.9	54.0±9.5
SV, ml	0.1±2.2	-4.2, 4.5	70.4±10.0	70.2±10.2	-0.2±4.5	-9.0, 8.6	63.8±9.5	64.0±10.8
**LV strain indices**								
3D-GLS, %	-0.4±1.6	-3.5, 2.6	21.7±2.3	21.2±1.5	-1.4± 2.2	-5.9, 3.0	21.0±2.3	19.5±2.5
GCS, %	-0.5±1.1	-2.7, 1.7	28.3±2.9	27.8±2.4	-0.4±2.0	-4.4, 3.7	25.9±2.2	25.5±2.1
Peak averaged CS, %	-0.4±1.2	-2.9, 2.0	28.5±2.8	28.1±2.4	-0.3±2.3	-4.9, 4.3	25.9±2.6	25.6±2.4
Peak averaged LS, %	-0.4±1.5	-3.5, 2.5	20.7±2.3	20.2±1.7	-1.2±2.3	-5.8, 3.4	20.3±2.0	19.1±2.7
Peak averaged PTS, %	-0.8±1.3	-3.4, 1.8	33.5±2.9	32.7±2.3	-1.4±2.8	-7.0, 4.1	32.2±2.9	30.7±2.7
Peak averaged RS, %	0.8±1.7	-2.5, 4.1	41.3±3.2	40.5±2.8	1.3±2.6	-3.9, 6.5	38.9±3.0	37.6±2.9
**LV rotational indices**								
Peak basal rotation,	0.0±0.2	-7.3, 7.3	8.0±4.0	8.0±3.9	-1.6±4.3	-10.0, 6.8	7.6±5.3	5.9±5.0
Peak apical rotation	0.2±2.9	-5.5, 5.9	6.4±2.5	6.2±2.9	0.47±3.6	-6.7, 7.7	4.9±2.7	4.4±3.3
Peak twist, °	0.1±6.4	-12.6, 12.7	13.7±5.7	13.7±6.1	2.1±7.0	-11.6, 15.9	11.9±6.4	9.7±7.5
Peak torsion, °/cm	0.0±0.8	-1.4, 1.4	1.6±0.6	1.6±0.7	0.3±0.8	-1.3, 1.8	1.4±0.7	1.1±0.8

Abbreviations: CS, circumferential strain; EDV, end-diastolic volume; EF, ejection fraction; ESV, end-systolic volume; GCS, global circumferential strain; GLS, global longitudinal strain; LOA, limits of agreement; LS, longitudinal strain; LV, left ventricular; PTS, principle tangential strain; RS, radial strain; SD, standard deviation; and SV, stroke volume.

Table S13. Intra-observer reproducibility based on re-reading the good quality scans (n=10) from the experimental poor technique study.

	Mean±SD		Bias (reading 1 – reading 2)	ICC
	1st reading	2nd reading	Mean Δ (95% CI)	
**LV volumetric indices**				
3D-EF, %	57.8±3.2	58.1±3.5	-0.3 (-0.1, 0.8)	0.97
EDV, ml	126.2±17.8	125.5±17.9	0.8 (-1.2, -0.3)	0.99
ESV, ml	53.5±9.9	52.8±10.1	0.7 (-1.2, -0.1)	0.99
SV, ml	72.7±9.5	72.7±9.9	-0.1 (-0.7, 0.6)	0.99
**LV strain indices**				
3D-GLS, %	21.2±2.5	20.5±2.7	0.7 (0.0, 1.3)	0.90
GCS, %	28.5±2.6	27.8±2.4	0.7 (0.1, 1.2)	0.93
Peak averaged CS, %	28.8±2.6	27.8±2.6	1.0 (0.4, 1.6)	0.91
Peak averaged LS, %	20.5±2.3	20.1±2.6	0.3 (-0.4, 1.1)	0.86
Peak averaged PTS, %	33.4±2.8	32.3±2.8	1.1 (0.4, 1.8)	0.91
Peak averaged RS, %	41.3±3.7	40.2±3.4	1.1 (0.5, 1.6)	0.96
**LV rotational indices**				
Peak basal rotation,	6.9±2.7	5.7±2.8	1.2 (0.3, 1.9)	0.86
Peak apical rotation	5.7±1.9	6.2±1.9	-0.5 (-1.0, -0.0)	0.89
Peak twist, °	11.6±3.9	11.3±4.3	0.3 (-0.7, 1.3)	0.91
Peak torsion,°/cm	1.3±0.4	1.3±0.5	0.1 (-0.1, 0.2)	0.92

Abbreviations: CS, circumferential strain; CI, confidence interval; EDV, end-diastolic volume; EF, ejection fraction; ESV, end-systolic volume; GCS, global circumferential strain; GLS, global longitudinal strain; LS, longitudinal strain; LV, left ventricular; PTS, principle tangential strain; RS, radial strain; SD, standard deviation; and SV, stroke volume.

Table S14. Inter-observer reproducibility based on re-reading the good quality scans (n=10) from the experimental poor technique study.

	Mean±SD		Bias (reading 1 – reading 2)	ICC
	1st reading	2nd reading	Mean Δ (95% CI)	
**LV volumetric indices**				
3D-EF, %	57.8±3.2	59.2±4.9	-1.4 (-3.3, 0.5)	0.71
EDV, ml	126.2±17.8	124.0±18.1	2.2 (1.2, 5.6)	0.95
ESV, ml	53.5±9.9	50.9±11.3	2.6 (0.3, 5.5)	0.89
SV, ml	72.7±9.5	73.1±9.9	-0.4 (-3.1, 2.3)	0.89
**LV strain indices**				
3D-GLS, %	21.2±2.5	21.6±2.4	-0.4 (-1.1, 0.3)	0.87
GCS, %	28.5±2.6	27.4±3.9	1.1 (-0.1, 2.3)	0.80
Peak averaged CS, %	28.8±2.6	28.3±4.0	0.4 (-0.8, 1.7)	0.79
Peak averaged LS, %	20.5±2.3	20.4±2.2	0.0 (-0.7, 0.8)	0.82
Peak averaged PTS, %	33.4±2.8	33.5±3.5	-0.1 (-1.5, 1.3)	0.73
Peak averaged RS, %	41.3±3.7	40.9±4.0	0.4 (0.6, 1.5)	0.89
**LV rotational indices**				
Peak basal rotation,	6.9±2.7	5.7±3.4	1.2 (-0.3, 2.7)	0.66
Peak apical rotation	5.7±1.9	5.3±2.6	0.4 (-0.7, 1.4)	0.69
Peak twist, °	11.6±3.9	10.2±5.8	1.4 (-1.2, 4.0)	0.59
Peak torsion,°/cm	1.3±0.4	1.1±0.6	0.2 (-0.1, 0.5)	0.60

Abbreviations: CS, circumferential strain; CI, confidence interval; EDV, end-diastolic volume; EF, ejection fraction; ESV, end-systolic volume; GCS, global circumferential strain; GLS, global longitudinal strain; LS, longitudinal strain; LV, left ventricular; PTS, principle tangential strain; RS, radial strain; SD, standard deviation; and SV, stroke volume.

Figure S1. Examples of impaired 3D echocardiographic (3DE) image quality.

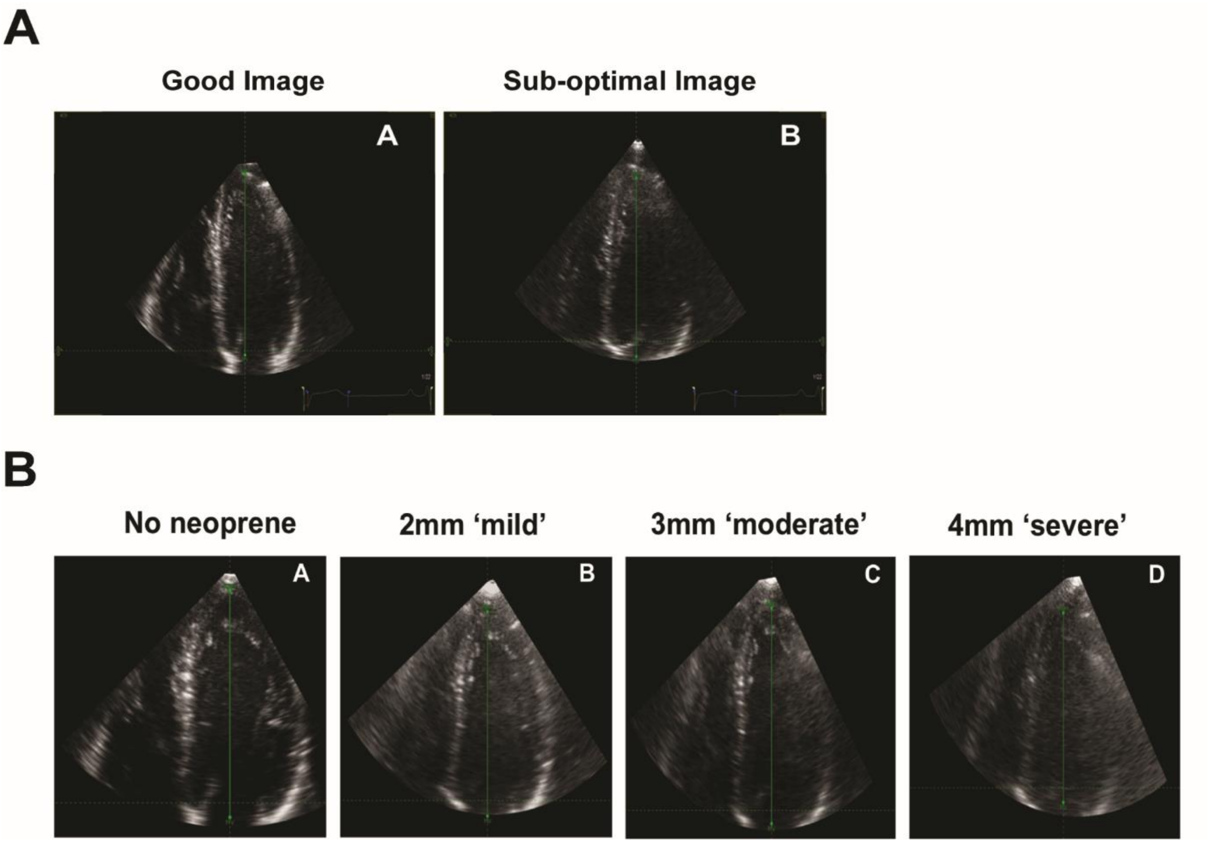

An example of a good and suboptimal 3DE image quality obtained from the same participant in the poor technique study**(A)**. An example of a 3DE with an optimal quality reference (no neoprene), mild (2mm neoprene), moderate (3mm neoprene), and severe (4mm neoprene) impairment of 3DE image quality obtained from the same participant in the neoprene study **(B)**.

Figure S2. Test-retest (scan re-scan) reliability.

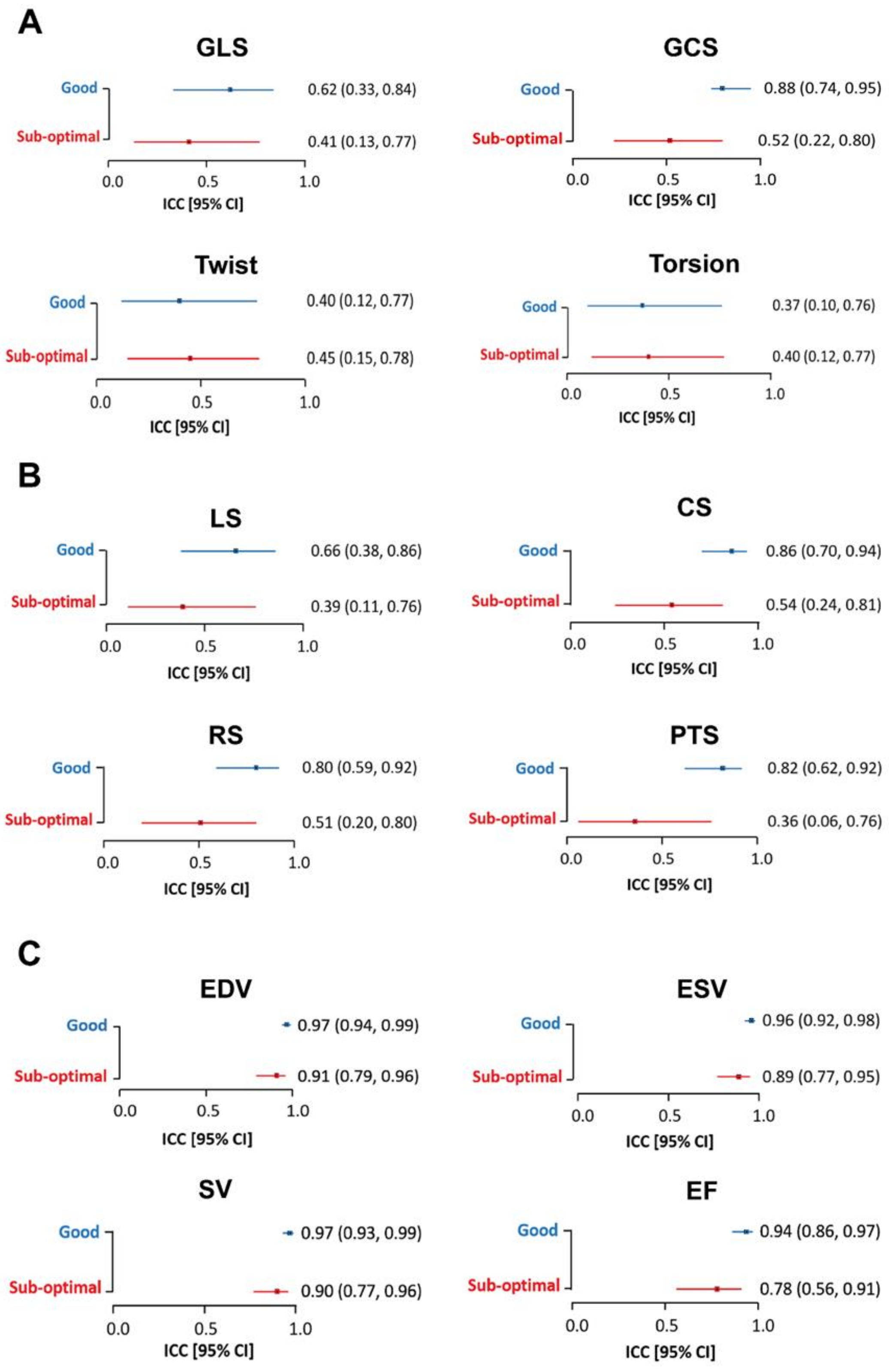

Intraclass correlation coefficient (ICC) of left ventricular (LV) global strain and rotational indices (A); peak averaged segmental LV strain indices (B); and volumetric indices (C). Good ICC represents the analysis of un-distorted quality images and sub-optimal ICC represents the analysis of distorted quality images. Abbreviations: CS, circumferential strain; CI, confidence interval; EDV, end-diastolic volume; EF, ejection fraction; ESV, end-systolic volume; GCS, global circumferential strain; GLS, global longitudinal strain; LS, longitudinal strain; PTS, principle tangential strain; RS, radial strain; and SV, stroke volume.

Figure S3. Bland & Altman Graphs.

A

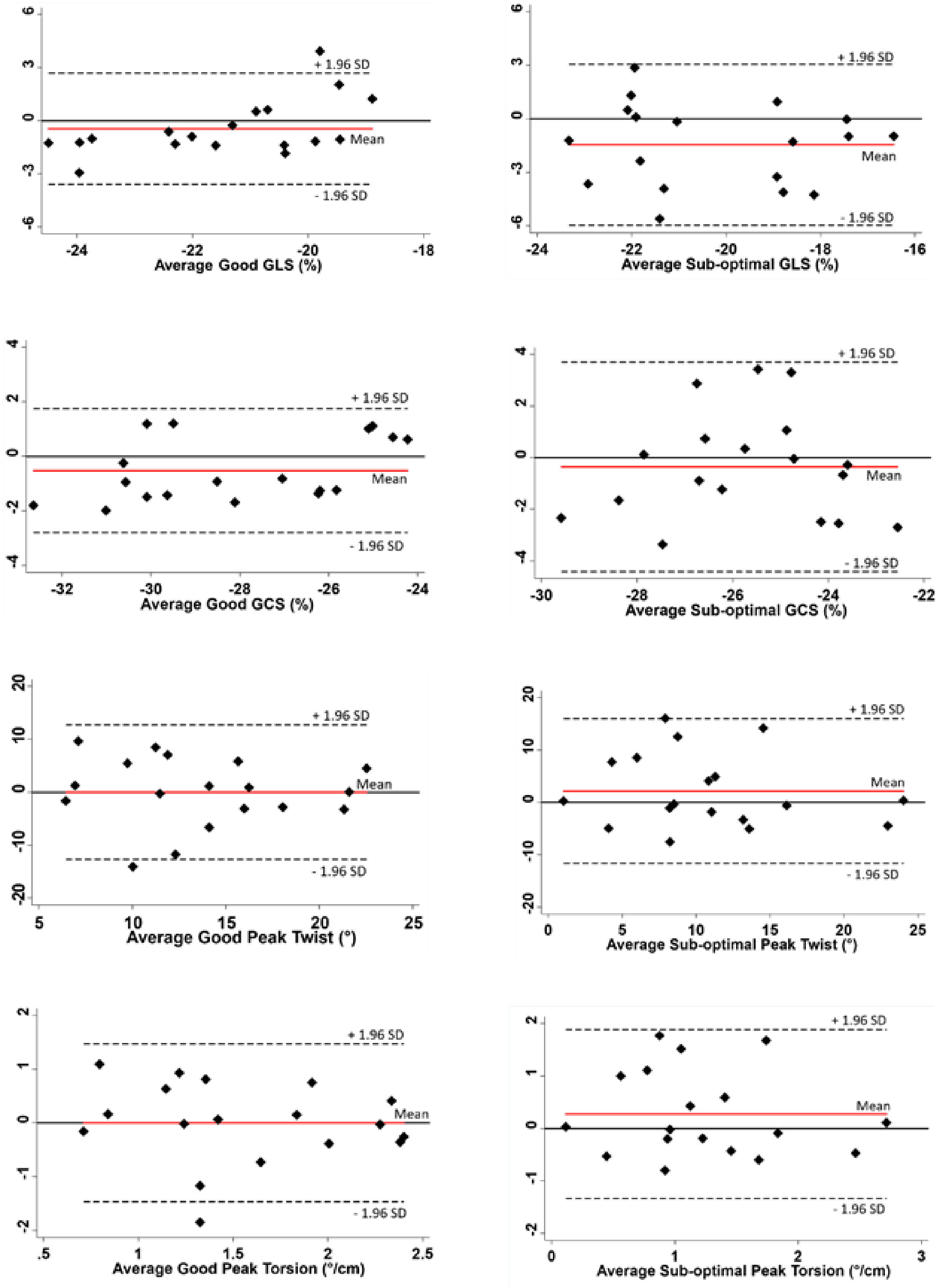

B

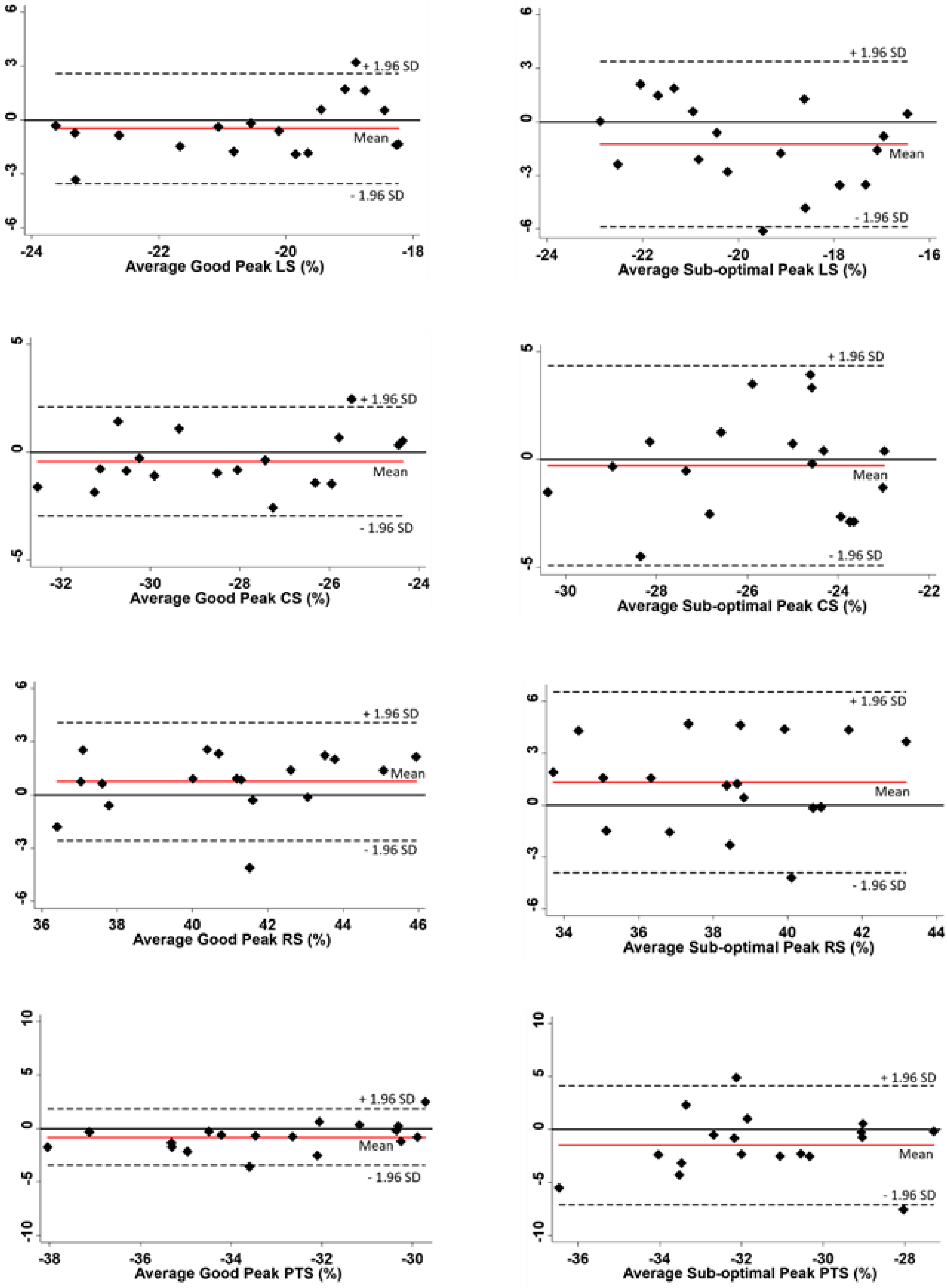

+ 1.96 SD
Mean
- 1.96 SD
Average Good Peak LS (%)
Average Sub-optimal Peak LS (%)
Average Good Peak CS (%)
Average Sub-optimal Peak CS (%)
Average Good Peak RS (%)
Average Sub-optimal Peak RS (%)
Average Good Peak PTS (%)
Average Sub-optimal Peak PTS (%)

C

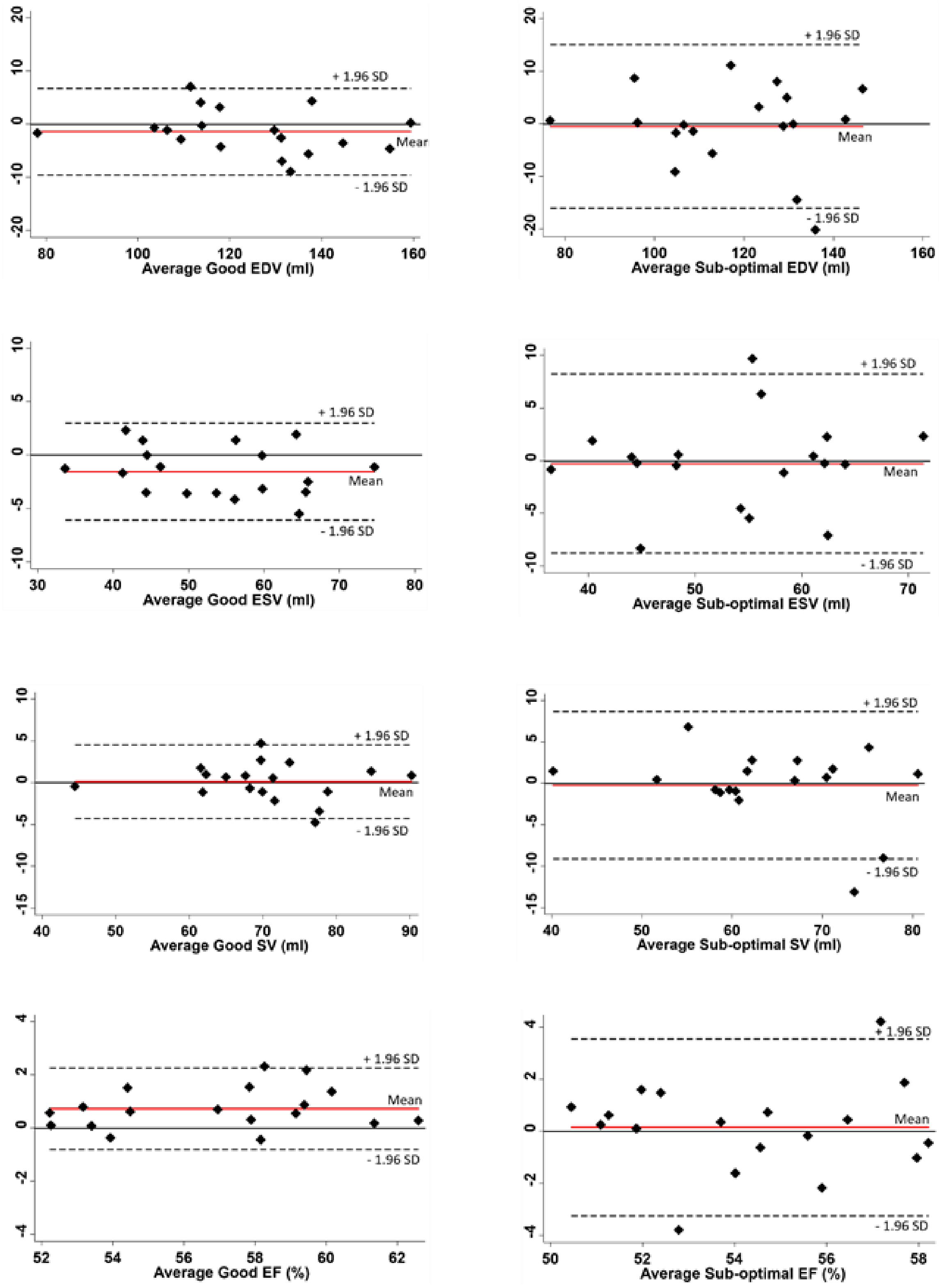

For these plots, actual strain not absolute strain values have been plotted of left ventricular (LV) global strain and rotational indices (A); peak averaged segmental LV strain indices (B); and volumetric indices (C).
